# Supplementary material for: Lack of abundant core virome in Culex mosquitoes from a temperate climate region despite a mosquito species-specific virome
Source: mSystems. 2024 May 14;9(6):e00012-24. doi: 10.1128/msystems.00012-24 (PMC11237611; doi:10.1128/msystems.00012-24)
Supplement: Supplemental material — Supplemental figures and tables. [file msystems.00012-24-s0001.pdf]

Supplementary tables

Supplementary Table S1

Virus	Primer	Sequence (5' → 3')
Daeseongdong virus 2	F	GAG CAT CCC TCG GGT TGA
	R	GAA CAC CCT GTT CAC CTT
	Probe	FAM-AAG TGT TTG AGG TGG CGA TGG AGA-TAMRA
Hubei mosquito virus 4	F	GCA ACC TCG ACT TCG TAT
	R	TGT GGA CAA ACA CCG GGT
	Probe	FAM-TCC CCG ATC TGC CGG TGT GTG-TAMRA
Culex orthophasmavirus 1	F	ACG GAC GCC AAT CAA TGA
	R	GCC TCT CAC CTG CAG AGT
	Probe	FAM-AGC TGT GCC GAC TTA ATG TTG GAA AGA-TAMRA
Wuhan mosquito virus 4	F	AGC GCA GTG ACA TTG ATT
	R	TAG GAC GGT CTC CAC AAC
	Probe	FAM-ACC ACC CTC GTC TGC ATT AAA TGG ATT G-TAMRA
Wuhan mosquito virus 6	F	GAC CCT GGA AAC TTT GTG
	R	AGC ACA GTC TCT GCA ACT TT
	Probe	FAM-CCA GAC CTT GAG ATG CCA GAA C-TAMRA
Xanthi chryso-like virus	F	CTT CAC CAT GTT GTC AGA
	R	ACT TTC GAA AGC CGA AGG
	Probe	FAM-ACC AGC GGG TGG AGG TAA AAC AAC A-TAMRA

Supplementary Table S2

Virus	Municipality	Species	Positive mosquitoes	Total mosquitoes	Infection rate (%)
Culex orthophasmavirus 2 (CPV)	Maasmechelen	*Culex pipiens pipiens*	1	24	4,17
	Vrasene	*Culex pipiens molestus*	18	24	75,00
		Culex pipiens pipiens	1	8	12,50
Daeseongdong virus 2 (DV2)	Bertem	*Culex pipiens pipiens*	9	13	69,23
	Eupen	*Culex pipiens pipiens*	7	24	29,17
		Culex torrentium	1	7	14,29
	Leuven	*Culex pipiens pipiens*	22	34	64,71
		Culex torrentium	3	6	50,00
	Maasmechelen	*Culex pipiens pipiens*	2	24	8,33
Hubei mosquito virus 4 (HMV4)	Maasmechelen	*Culex pipiens pipiens*	2	24	8,33
	Villers-Le-Bouillet	*Culex pipiens pipiens*	6	17	35,29
	Vrasene	*Culex pipiens molestus*	1	24	4,17
		Culex pipiens pipiens	4	8	50,00
Wuhan Mosquito Virus 4 (WMV4)	Bertem	*Culex pipiens pipiens*	1	13	7,69
	Dilsen-Stokkem	*Culex pipiens molestus*	2	4	50,00
	Eupen	*Culex pipiens pipiens*	1	24	4,17
	Leuven	*Culex pipiens pipiens*	4	34	11,76
	Maasmechelen	*Culex pipiens pipiens*	2	24	8,33
	Vrasene	*Culex pipiens molestus*	10	24	41,67
Wuhan Mosquito Virus 6 (WMV6)	Bertem	*Culex pipiens pipiens*	2	13	15,38
	Frameries	*Culex pipiens molestus*	1	2	50,00
	Kallo	*Culex pipiens pipiens*	1	1	100,00
	Leuven	*Culex pipiens pipiens*	2	34	5,88
	Maasmechelen	*Culex torrentium*	1	2	50,00
	Natoye	*Aedes japonicus*	1	8	12,50
	Villers-Le-Bouillet	*Culex pipiens pipiens*	1	17	5,88
	Vrasene	*Culex pipiens molestus*	3	24	12,50
		Culex pipiens pipiens	1	8	12,50
Xanthi chryso-like virus (XCV)	Bertem	*Culex pipiens pipiens*	2	13	15,38
	Eupen	*Culex pipiens pipiens*	1	24	4,17
	Frameries	*Culex pipiens molestus*	1	2	50,00
		Culex pipiens pipiens	3	5	60,00
	Leuven	*Culex pipiens pipiens*	6	34	17,65
	Maasmechelen	*Culex pipiens pipiens*	2	24	8,33
	Villers-Le-Bouillet	*Culex pipiens pipiens*	3	17	17,65
	Vrasene	*Culex pipiens molestus*	1	24	4,17
		Culex pipiens pipiens	2	8	25,00

Supplementary Table S3

Virus	Species	Positive mosquitoes	Total mosquitoes	Infection rate (%)
Culex orthophasmavirus 2 (CPV)	*Aedes japonicus*	0	8	0,00
	Culex pipiens molestus	18	47	38,30
	Culex pipiens pipiens	2	127	1,57
	Culex torrentium	0	16	0,00
Daeseongdong virus 2 (DV2)	*Aedes japonicus*	0	8	0,00
	Culex pipiens molestus	0	47	0,00
	Culex pipiens pipiens	40	127	31,50
	Culex torrentium	4	16	25,00
Hubei mosquito virus 4 (HMV4)	*Aedes japonicus*	0	8	0,00
	Culex pipiens molestus	1	47	2,13
	Culex pipiens pipiens	12	127	9,45
	Culex torrentium	0	16	0,00
Wuhan Mosquito Virus 4 (WMV4)	*Aedes japonicus*	0	8	0,00
	Culex pipiens molestus	12	47	25,53
	Culex pipiens pipiens	8	127	6,30
	Culex torrentium	0	16	0,00
Wuhan Mosquito Virus 6 (WMV6)	*Aedes japonicus*	1	8	12,50
	Culex pipiens molestus	4	47	8,51
	Culex pipiens pipiens	7	127	5,51
	Culex torrentium	1	16	6,25
Xanthi chryso-like virus (XCV)	*Aedes japonicus*	0	8	0,00
	Culex pipiens molestus	2	47	4,26
	Culex pipiens pipiens	19	127	14,96
	Culex torrentium	0	16	0,00

Supplementary figures

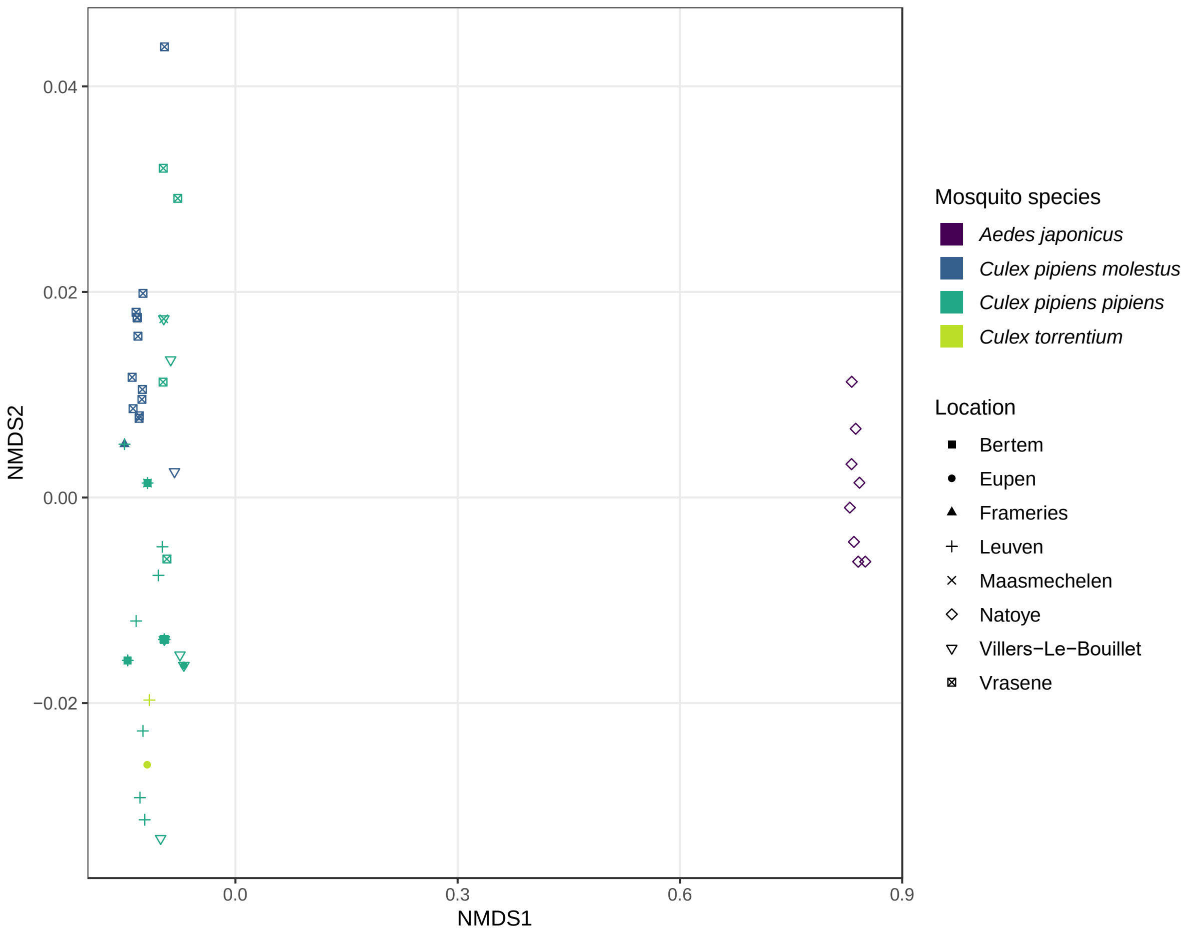

Supplementary Figure S1. NMDS plot of the mosquito viromes with removed singleton samples.

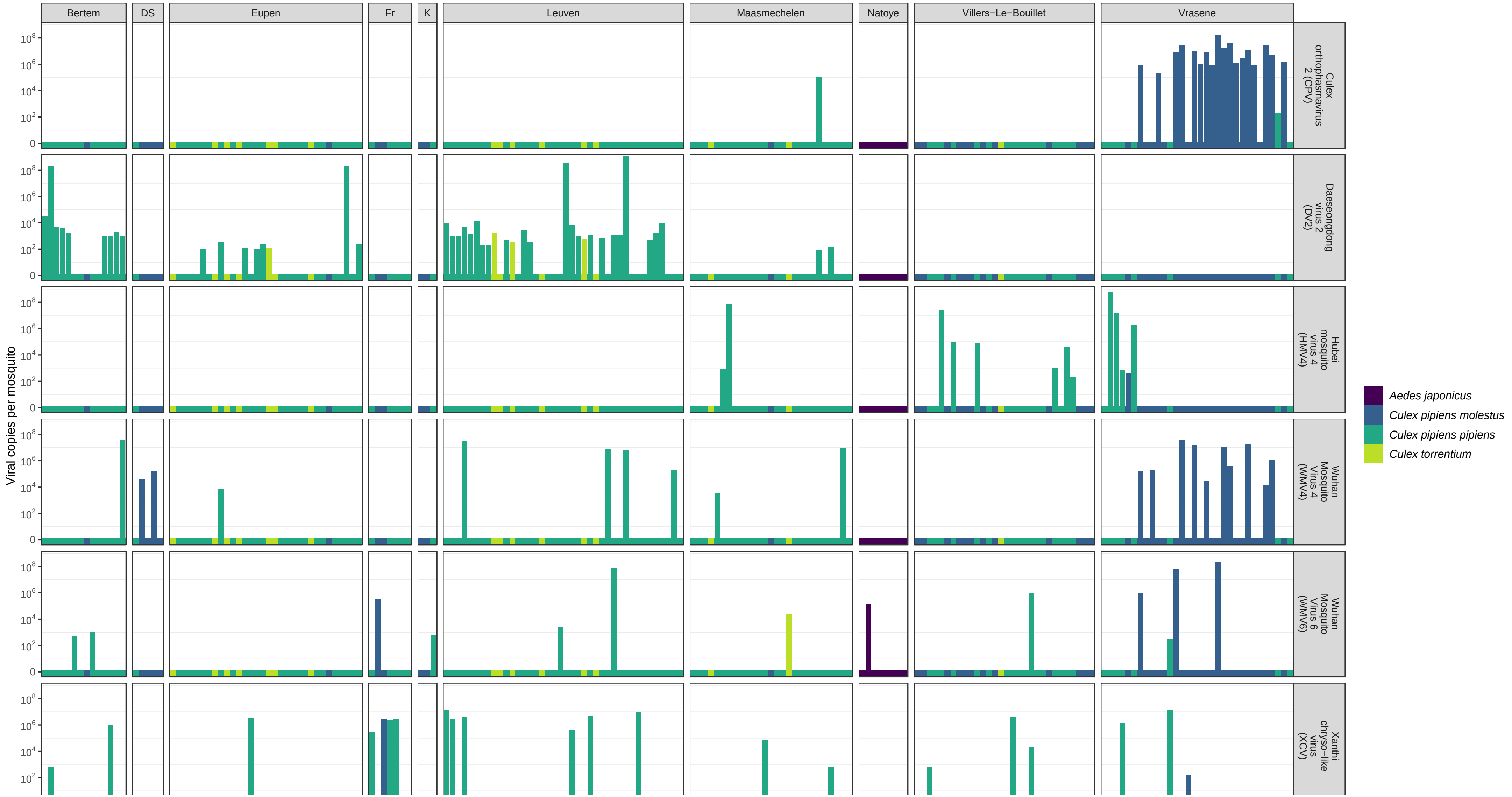

Supplementary Figure S2. qPCR genome copy numbers per individual mosquito sorted by virus and location. (DS=Dilsen-Stokkem, Fr=Frameries, K=Kallo)

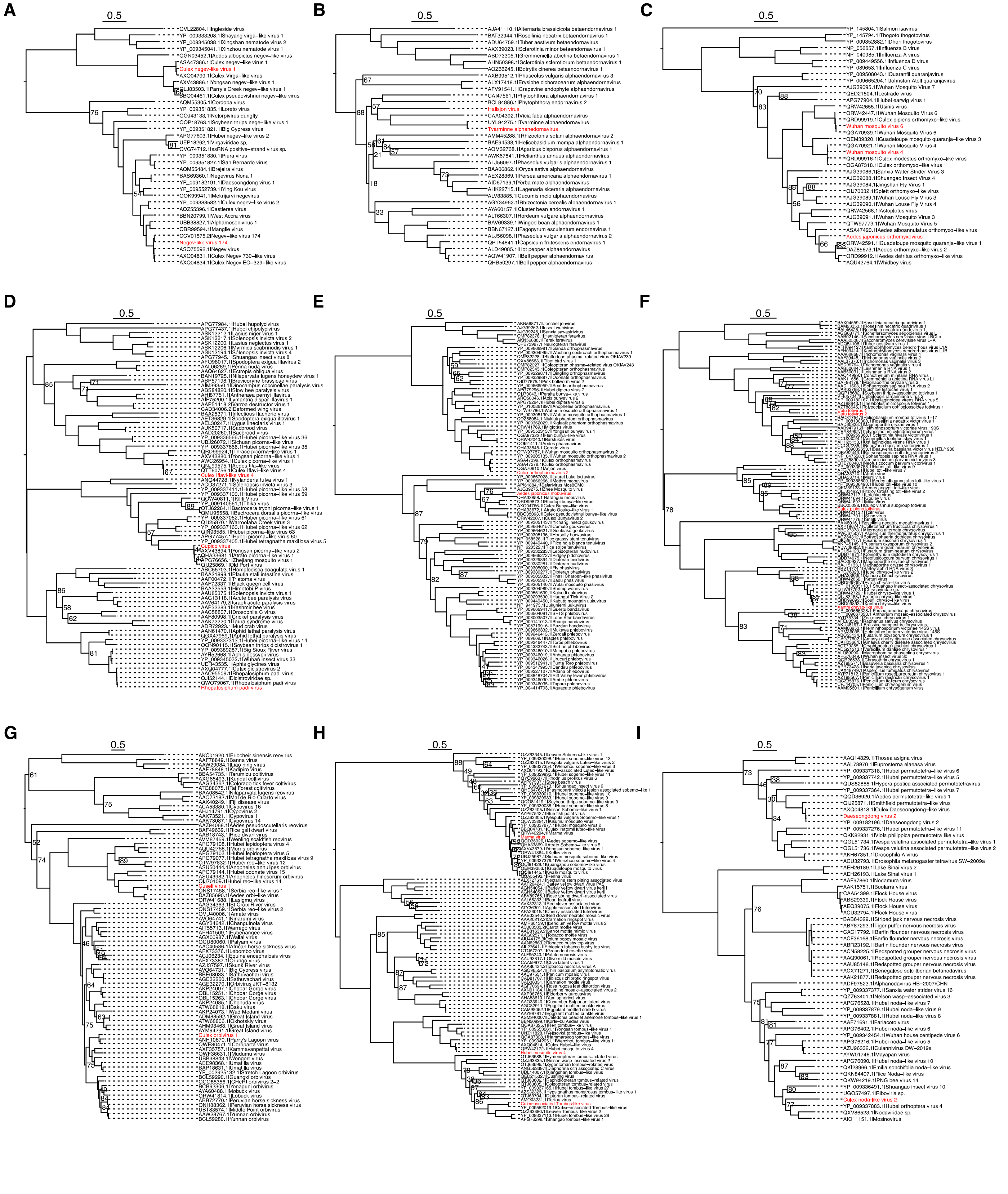

Supplementary Figure S3. Phylogenetic trees with accession numbers and virus names.

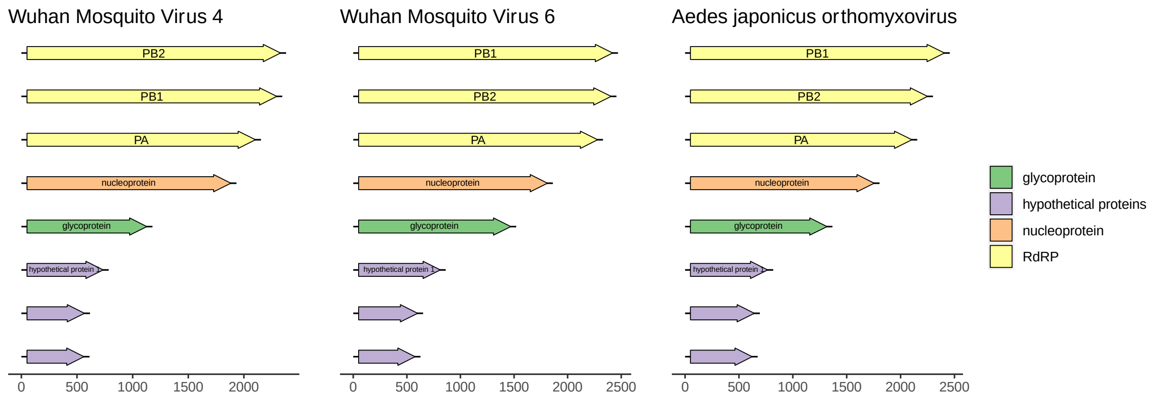

Supplementary Figure S4. Genome layout of the orthomyxoviruses identified in this study.

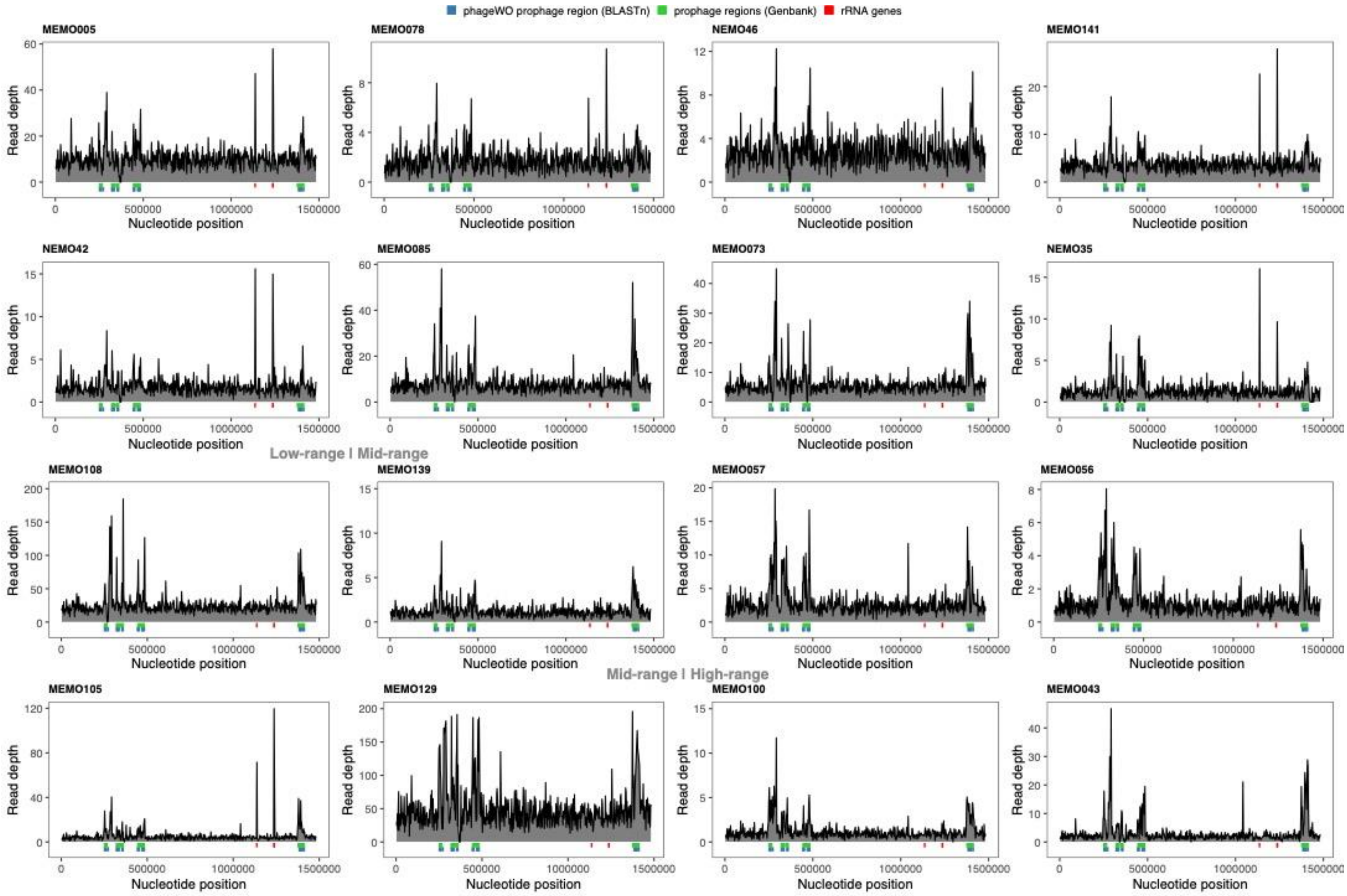

Supplementary Figure S5. Coverage plots of low-, mid- and high-range depth ratio samples.

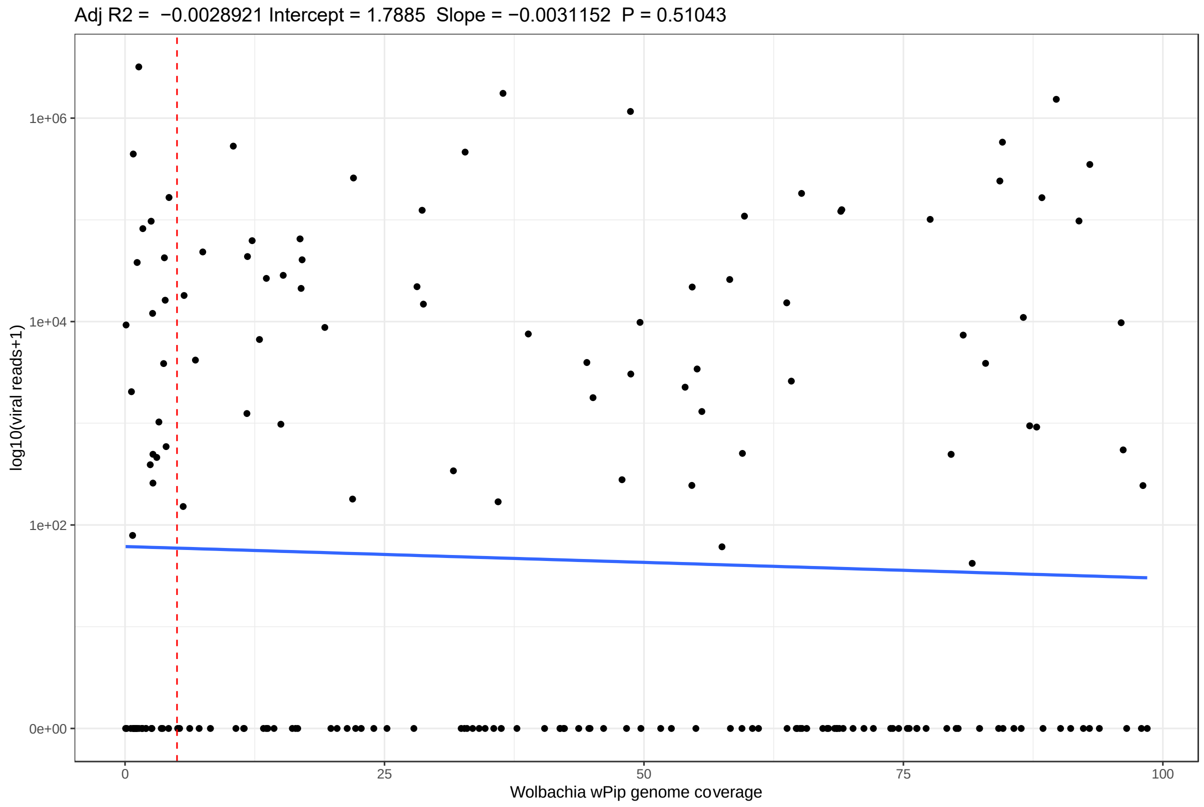

Supplementary Figure S6. Correlation between presence of Wolbachia and viral reads. Wolbachia was considered present if the genome coverage was larger than 5%.

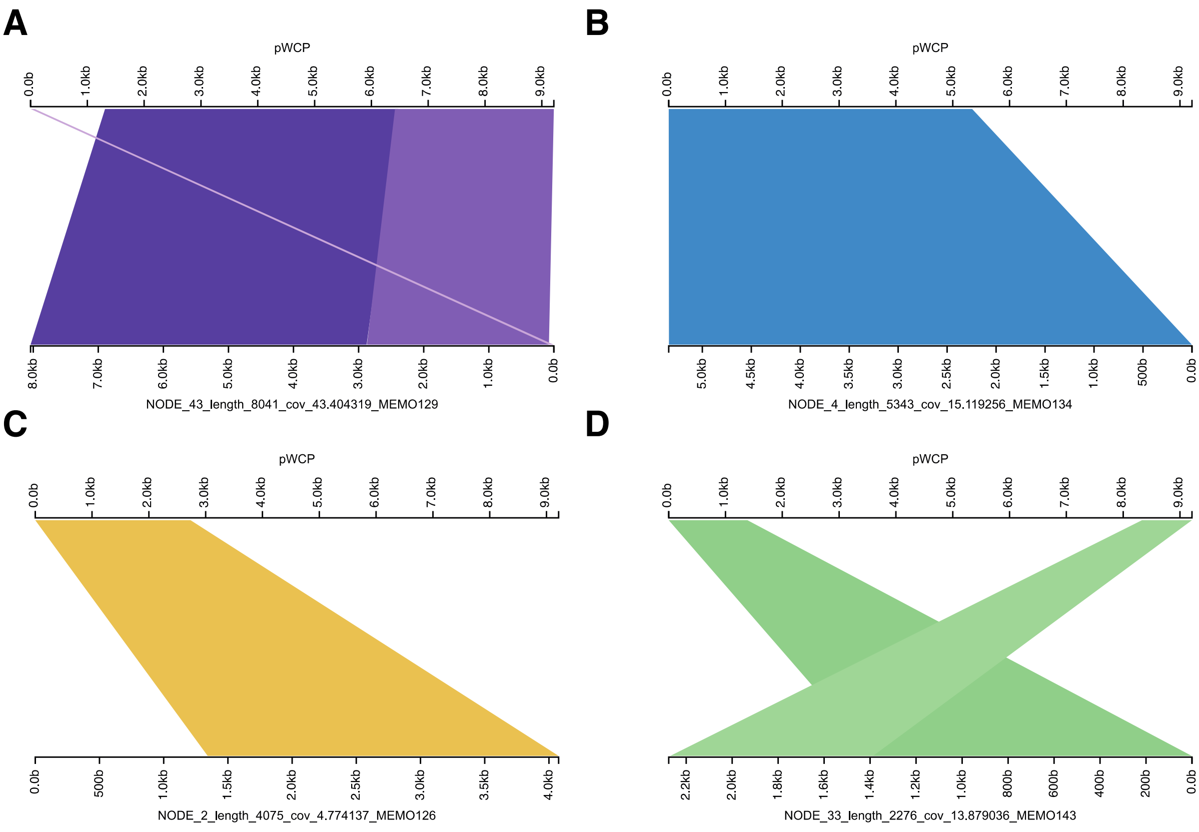

Supplementary Figure S7. Alignments between the pWCP plasmid reference and contigs of our study.
